# Intravenous Methylprednisolone in Induction Therapy for ANCA-Associated Vasculitis: How Low Can We Go?

**DOI:** 10.34067/KID.0000000000000257

**Published:** 2023-09-28

**Authors:** Nestor Oliva-Damaso, Andrew S. Bomback

**Affiliations:** 1 Division of Nephrology, Department of Medicine, Hospital Costa del Sol, Marbella, Spain; 2 Division of Nephrology, Department of Medicine, Columbia University Irving Medical Center, New York, New York

**Keywords:** ANCA-associated vasculitis, steroids toxicity, intravenous methylprednisolone pulses

ANCA-associated vasculitides (AAVs) are a group of clinical entities that includes granulomatosis with polyangiitis, microscopic polyangiitis, and eosinophilic granulomatosis with polyangiitis, characterized by necrotizing inflammation of small-sized and medium-sized blood vessels due to inflammatory cell infiltration directed against two main antigenic targets: myeloperoxidase and leukocyte proteinase 3.^1,2^ Administration of intravenous (IV) methylprednisolone (MTP) pulses, in doses of 1000–3000 mg over 3 days, has been used in induction protocols of the main trials of AAVs, including the Plasma Exchange and Glucocorticoids for the Treatment of ANCA-Associated Vasculitis (PEXIVAS), Plasma Exchange for Renal Vasculitis (MEPEX), Rituximab in ANCA-Associated Vasculitis (RAVE), and others,^2^ and is also a common practice in many institutions, without an evidence base. Specifically, no head-to-head trials have studied the role of MTP pulses in AAVs, the best available evidence being derived from indirect comparison across different trials.^2,3^

In this issue of *Kidney360*, Floyd *et al.* present a single-center, retrospective cohort study of patients newly diagnosed with AAVs, including severe or life-threatening disease, treated with induction regimens of cyclophosphamide, rituximab, or combination therapy of both on the basis of physician discretion. Their analysis compared 34 patients who received high-dose GC induction treatment (1.5 g IV MTP over 3 days, followed by oral prednisolone starting at 40–60 mg per day) and 31 who received low-dose GC therapy (single dose of 250 mg pulsed IV MTP, followed by oral prednisolone starting at 30 mg per day). The primary outcome was a composite of death and ESKD while secondary outcomes included remission, relapses, and serious adverse events. ESKD was similar between groups at 6 and 12 months. More deaths occurred in the high-dose group (26.5% versus 6.5%, *P* = 0.05), although on multivariable analysis this was not significant (*P* = 0.06). Remission rates were comparable, and there was no significant difference in relapses. Adverse events were seen in both groups, but the high-dose patients experienced a higher number of severe infections, weight gain, and steroid-induced diabetes. The authors concluded that the markedly reduced dosing of IV MTP used in their low-dose GC cohort was safe and effective compared with conventional IV MTP dosing.

The cumulative mean GC dose at 12 months of high-dose GC induction treatment was 5604±1168 mg compared with 2893±536 mg in the low-dose GC group. With the inherent limitations of a retrospective analysis, this study nonetheless aligns with other observational studies that have reported no efficacy benefit but increased rates of infections with the use of higher initial doses of GC, including MTP pulses.^2^ Other retrospective studies evaluated the feasible strategy of IV MTP avoidance where pulse MTP did not identify any benefit on patient survival, renal survival, or 12-month relapse rates but did confirm a higher rate of treatment-related adverse effects, including infection and diabetes.^4,5^ The Low-Dose GC Vasculitis Induction Study,^6^ a randomized clinical trial sampling nonsevere disease (without severe glomerulonephritis or alveolar hemorrhage and a predominance of myeloperoxidase-ANCA vasculitis), compared a low and high oral GC dosing (0.5 versus 1 mg/kg daily) alongside rituximab, *in the absence of IV MTP at induction*. The low-dose group yielded noninferior results compared with the high-dose group. In another retrospective study^7^ that included patients with severe disease (diffuse alveolar hemorrhage or severely impaired GFRs), the addition of pulsed IV GC to plasma exchange did not improve early patient or kidney survival but was associated with a greater risk of adverse events.

The Plasma Exchange and Glucocorticoids for the Treatment of ANCA-Associated Vasculitis trial demonstrated that for patients with eGFR <50 ml/min per 1.73 m^2^, a more rapid reduction was as effective but safer than a standard glucocorticoid (GC) tapering regimen,^8^ although pulses of MTP 1–3 g were used in this trial. The Avacopan in Patients with ANCA-Associated Vasculitis study posited avacopan, a C5a receptor blocker, as an effective alternative to GC treatment in AAVs.^9^ Patients with severe disease (eGFR <15 ml/min per 1.73 m^2^ and/or alveolar hemorrhage requiring mechanical ventilation) were excluded, but remission at week 26 was observed in 72.3% of patients in the avacopan arm and 70.1% in the prednisolone arm, achieving noninferiority. Still, even those in the avacopan (steroid-free) arm were allowed to receive a cumulative dose up to 3 g MTP equivalent in the 4-week period before screening. Both studies facilitate steroids avoidance strategies and point GC as major contributors to adverse events without direct information of IV MTP induction therapy where the optimal dose of pulse is yet to be determined.

The novelty of the study by Floyd *et al.* in this issue of *Kidney360* is its focus on reductions in the pulse MTP dosing that begins induction therapy and, to date, has not been challenged even in low-dose or steroid-free regimens (this applies not just to AAVs but other forms of progressive glomerulonephritides, including lupus nephritis and crescentic IgA nephropathy). GCs—*IV pulse and oral*—continue to be part of the standard of care of patients with glomerular diseases, such as AAVs,^2,3^ but recommendations are moving quickly toward reducing oral GC dosing given the increasing evidence that adverse events can be reduced with lower GC dosing without losing efficacy. Indeed, patients with an increased risk of GCs toxicity are likely to have the most benefit from low-dose steroid regimens or steroid substitutes, such as avacopan.^3,9^ The use of the GC toxicity index (GTI) provides a global, nuanced quantifiable assessment tool in which clinicians can assess GC-associated morbidity. The GTI includes domains, such as BMI, blood pressure, glucose tolerance, lipid metabolism, GC myopathy, skin toxicity, neuropsychiatric effects, and infections. However, studies of the use of pulsed IV MTP and GTI score (or other measures of steroid toxicity) in AAVs remain scarce.^10^

High-dose IV GCs with MTP (doses of 1–3 g given over 72 hours) are routinely used in severe presentations of AAVs, such as rapidly progressive glomerulonephritis, pulmonary hemorrhage, mononeuritis multiplex, or optic neuritis. However, this dosing has not been tested in randomized controlled trials. The contribution of high-dose IV MTP to the cumulative dose of GC is enormous, as highlighted in the study by Floyd *et al.*, and likely influences the overall toxicity profile of GC. Theirs, however, is not destined to be the study that changes how we use pulse MTP in the treatment of ANCA given that the analysis is based on retrospective data with treatment assignments ultimately determined by physician discretion. The possibility of selection bias—specifically patients with milder disease being given the lower IV MTP dosing—cannot be overlooked. Their results, however, clearly call for further research on this topic. We have already seen a number of important and well-designed studies in AAVs and other glomerular diseases that have shown that we can use the lower dose of oral GC therapy. The study by Floyd *et al.* establishes the need for future randomized clinical trials evaluating different pulses of MTP regimens across a wide range of clinical presentations of ANCA-associated disease.
